# Flexible oxygen concentrators for medical applications

**DOI:** 10.1038/s41598-021-93796-3

**Published:** 2021-07-12

**Authors:** Akhil Arora, M. M. Faruque Hasan

**Affiliations:** Artie McFerrin Department of Chemical Engineering, Texas A&M University, College Station, TX 77843-3122 USA

**Keywords:** Chemical engineering, Health services, Quality of life

## Abstract

Medical oxygen concentrators (MOCs) are used for supplying medical grade oxygen to prevent hypoxemia-related complications related to COVID-19, chronic obstructive pulmonary disease (COPD), chronic bronchitis and pneumonia. MOCs often use a technology called pressure swing adsorption (PSA), which relies on nitrogen-selective adsorbents for producing oxygen from ambient air. MOCs are often designed for fixed product specifications, thereby limiting their use in meeting varying product specifications caused by a change in patient’s medical condition or activity. To address this limitation, we design and optimize flexible single-bed MOC systems that are capable of meeting varying product specification requirements. Specifically, we employ a simulation-based optimization framework for optimizing flexible PSA- and pressure vacuum swing adsorption (PVSA)-based MOC systems. Detailed optimization studies are performed to benchmark the performance limits of LiX, LiLSX and 5A zeolite adsorbents. The results indicate that LiLSX outperforms both LiX and 5A, and can produce 90% pure oxygen at 21.7 L/min. Moreover, the LiLSX-based flexible PVSA system can manufacture varying levels of oxygen purity and flow rate in the range 93–95.7% and 1–15 L/min, respectively. The flexible MOC technology paves way for transitioning to an envisioned cyber-physical system with real-time oxygen demand sensing and delivery for improved patient care.

## Introduction

Portable medical oxygen concentrators (MOCs) have found wide use in facilitating home-based oxygen therapy for patients suffering from lung conditions including COVID-19, chronic obstructive pulmonary disease (COPD), chronic bronchitis and pneumonia, among others. To produce pure oxygen from ambient air at small scales, several adsorbent technologies based on pressure swing adsorption (PSA) have been commercialized^1–3^. Therefore, a majority of MOCs rely on a PSA process with a nitrogen-selective adsorbent. According to the World Health Organization (WHO), medical-grade oxygen has oxygen concentration between 90 and 96% V/V with remaining nitrogen and argon. In alignment with these specifications, the typical oxygen product obtained from adsorption-based MOC devices consists of 90–93% oxygen at a production rate of less than 10 L/min^4^.

In adsorption-based MOCs, due to limited adsorption capacity, the adsorbent is periodically regenerated for efficient utilization. To facilitate continuous oxygen supply, either the product oxygen can be collected in a surge column and supplied at a constant time-averaged rate, or a multi-bed operation can be utilized. Skarstrom-type PSA cycle configuration is typically utilized in MOCs, which consists of production, depressurization, purge and pressurization steps. Based on the pressure levels of production and purge steps, three different subclasses of PSA exist that is pressure swing adsorption, vacuum swing adsorption (VSA) and pressure vacuum swing adsorption (PVSA)^4^. MOCs leverage rapid cycling of adsorption column to maximize adsorbent utilization and miniaturize the size of the operation^5^. In addition, small adsorbent particle sizes are used to reduce the mass transfer resistances and enhance the adsorption kinetics.

**Figure 1:**
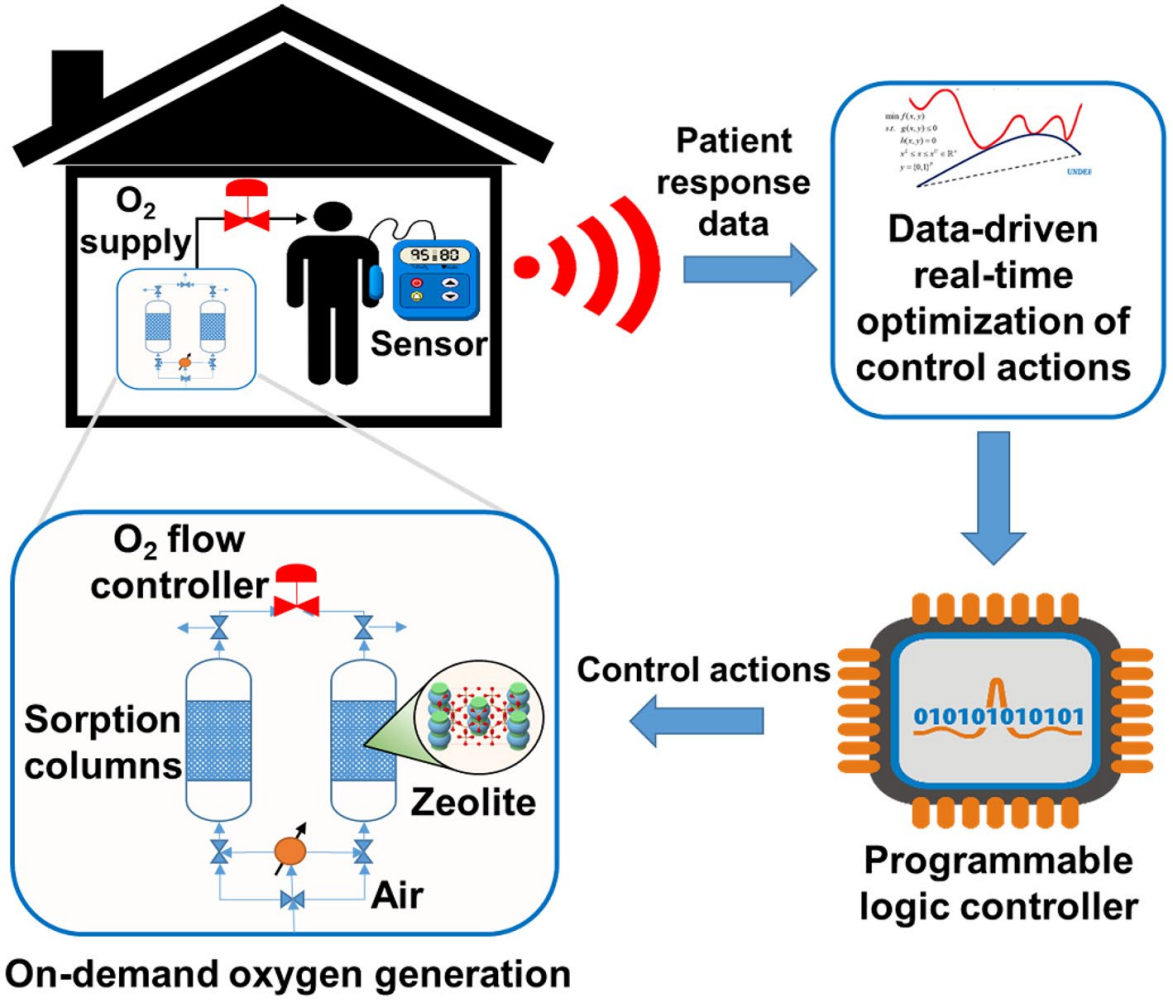
Envisioned cyber-physical system for online oxygen demand sensing and home-based delivery using adsorption-based medical oxygen concentrator (MOC).

For medical use, depending on the condition of end-use patients and whether the patient is at rest or active, the required specifications of oxygen product could vary both in terms of flow rate and purity^6^. In addition, the same oxygen concentrator unit can be used for several different patients in a hospital setting. Therefore, it is desirable to design a flexible and modular PSA process that can rapidly switch between different operating regimes for on-demand oxygen production while fulfilling different product specifications. To meet the time-varying oxygen demand, we envision a cyber-physical system (CPS) within which the blood oxygen concentration of a patient suffering from a lung condition is constantly monitored, and necessary actions to modify the operation of MOC are taken in real-time (Fig. 1).

In the envisioned CPS, a blood oxygen saturation sensor transmits the recorded data to a controller. The controller checks the patient’s status, and if there is a requirement of modifying oxygen specifications due to a change in patient’s health condition or activity, optimal control actions are determined and transmitted to the MOC. The MOC is accordingly reconfigured to adjust oxygen production flow rate and purity. Within this real-time data-based flexible PSA operation, one of the most challenging tasks is to determine the optimal control action policies, and predict the dependence of outlet product specifications on input control actions. Specifically, it is challenging to optimally design and operate PSA columns due to their inherent nonlinear dynamics, complex process operation and variable operating regimes. For optimal operation, several decision variables need to be evaluated which include cycle configuration and operation, pressure levels, purging conditions and bed regeneration efficiency. In addition, there are several objectives that need to be met which include modularity, compactness, reliability and efficiency.

Some of the most crucial metrics that are used for judging the performance of a PSA-based MOC are bed size factor (BSF) and oxygen recovery^4^. BSF is computed by obtaining the amount of adsorbent required to produce 1 ton of oxygen per day (TPD), and is represented with the unit kg ads. $\hbox {O}_{2}$ TPD$^{-1}$. Therefore, minimizing the BSF leads to lower adsorbent inventory levels and smaller MOC units. On the other hand, oxygen recovery is computed by calculating the fraction of oxygen recovered in the product outlet relative to the amount of oxygen fed during a PSA cycle at a cyclic steady state. Consequently, for a given product specification, a higher oxygen recovery leads to lower compression costs and lower ambient air feed flow rates. As MOC is a small-scale device with limited adsorbent amount and rapid cycling, there is a high energy consumption due to frequent pressure variation as compared to conventional PSA operation. However, for small-scale applications, the relative simplicity and reliability of MOC play a more significant role as compared to energy consumption^3^. Overall, the key design goals while developing PSA-based MOC are (1) increasing adsorbent productivity, (2) enhancing oxygen recovery and (3) developing compact and lightweight units^7^.

To meet the aforementioned processing objectives, several literature studies focus on using different adsorption cycles and materials for performing air separation. Table 1 summarizes the process characteristics and performance metrics for different literature studies. Farooq et al.^8^ performed simulation and experimental studies to investigate a 2-bed 4-step PSA process for air separation using 5A zeolite. The theoretical results showed that oxygen product with 93.4% purity can be obtained, albeit at a low oxygen recovery of 20.1% and a low production rate of 0.07 L/min. To increase the air separation efficiency, Kopaygorodsky et al.^9^ evaluated the viability of ultra-rapid PSA cycles using 5A zeolite. It was observed that 85% oxygen product with a product recovery of 60% could be obtained with a total cycle time less than 3 seconds and for a small BSF of 0.0073, thereby leading to more compact and efficient PSA units. Santos et al.^10^ performed simulation and optimization studies for studying 4-step PSA and PVSA cycles for oxygen production with three different candidate adsorbents, i.e., Oxysiv 5, Oxysiv 7 and SYLOBEAD MS S 624. Their analysis indicated that Oxysiv 7 had the best separation performance for both PSA and PVSA cycles with 94.5% oxygen purity, 21.3% recovery and 3.7 L/min production rate. They further extended their analysis to investigate a 6-step PSA cycle for small-scale medical applications, and obtained a 94.5% pure oxygen product with 34.1% recovery and 4.3 L/min production rate^11^.

**Table 1:** Literature studies on adsorption-based air separation for medical grade oxygen production.

Authors	Adsorbent	Cycle type and duration	Operating pressures (bar)	Process performance			
				Purity (%)	Recovery (%)	Flow rate (L/min)	BSF (kg ads. $\hbox {O}_{2}$ TPD$^{-1}$)
Farooq et al.^8^	5A zeolite	4-step PSA, 100 s	$P_a$ = 1.5, $P_d$ = 1.01	93.4	20.1	0.07	–
Kopaygorodsky et al.^9^	5A zeolite	2-step PSA, < 3 s	$P_a$ = 1.52, $P_d$ = 1.01	85	56	–	0.0073
Santos et al.^10^	Oxysiv 5, 7&	4-step PSA, 18 s	$P_a$ = 3, $P_d$ = 1	94.5	21.3	3.7	–
	Sylobead MS S 624						
Santos et al.^11^	Oxysiv 5, 7&	6-step PSA, 16 s	$P_a$ = 3, $P_d$ = 1	94.5	34.1	4.3	–
	Sylobead MS S 624						
Rao et al.^12^	5A & Ag-Li-X zeolite	2-step PSA, 1.32 s	$P_a$ = 3.55, $P_d$ = 1.01	90	10–55	5	–
Chai et al.^4^	LiX zeolite	4-step PSA, 3–5 s	$P_a$ = 3.04–4.05, $P_d$ = 1.01	90	25–35	5	11.3–22.7
Rao et al.^7^	LiLSX zeolite	4-step PSA, 3–9 s	$P_a$ = 4, $P_d$ = 1	90	15-30	1–3	45–70
Zhu et al.^13^	LiLSX zeolite	5-step PVSA, 7 s	$P_a$ = 2.4, $P_d$ = 0.6	90	29.5	0.75	82.8
Moran et al.^14^	LiLSX zeolite	2-bed 6-step PVSA, 3.8–6.8 s	$P_a$ = 1.95, $P_d$ = 0.43	92–93	41–45	–	23.1–36.7
Zhu et al.^15^	LiLSX zeolite	4-bed 6-step PSA, 5 s	$P_a$ = 2.53, $P_d$ = 1.01	92	30	1	78

Rao et al.^12^ designed a 2-step pulsed PSA process to evaluate the extent of miniaturization possible for medical applications. With alternating pressurization and depressurization steps, they were able to achieve an oxygen purity of 90% at a production rate of 5 L/min using 5A and Ag-Li-X zeolite adsorbents. Rao et al.^7^ further developed a 4-step rapid PSA process which produces 90% oxygen product at 1–3 L/min with an oxygen recovery of 15–30% and BSF of 45–70. Chai et al.^4^ developed a rapid PSA process using LiX zeolite with a total cycle time in the range 3–5 s, and were able to achieve 90% pure oxygen product with 25–35% recovery and a BSF of 11.3–26.7 kg ads. $\hbox {O}_{2}$ TPD$^{-1}$. Zhu et al.^13^ devised a rapid PVSA process with intermediate pressurization steps using LiLSX zeolite and observed that lower desorption pressure levels help in lowering BSF and increasing oxygen recovery. The developed test unit produced 90% pure oxygen at 0.75 L/min with a BSF of 82.8 kg ads. $\hbox {O}_{2}$ TPD$^{-1}$ and an oxygen recovery of 29.5%. More recently, they developed a 4-bed rotary value rapid PSA process to enhance the air separation performance^15^. The results indicated that an oxygen purity of 92% at 1 L/min production can be achieved with an oxygen recovery of 30% and a BSF of 78.

A majority of the existing literature studies predominantly focuses on designing PSA-based medical oxygen generation technologies for fixed product specifications. However, designing flexible PSA processes that can cater to a variety of end-use oxygen specifications is desirable for repurposing a flexible MOC unit to suit different product and/or patients’ requirements. Even though there exists plenty of literature in the domain of optimization-based flexible process design and operation for chemical engineering systems^16–19^, very few studies perform flexibility analysis for complex systems such as that of PSA.

**Figure 2:**
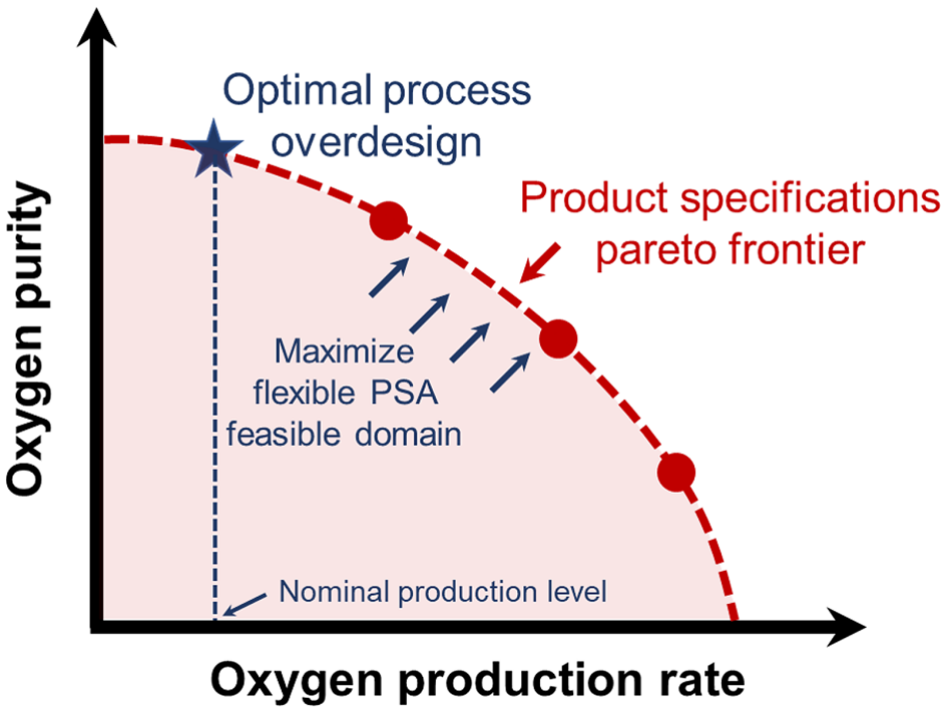
An illustrative depiction of product specifications Pareto frontier and feasible domain for pressure swing adsorption (PSA)-based flexible MOC operation. The key objective is to push the Pareto frontier and maximize the feasible PSA operation area using the simulation-based optimization framework.

To this end, we study the operational flexibility of PSA-based MOC systems and map the effects of different design and operating conditions on outlet product specifications. For this analysis, we use an in-house simulation-based optimization framework, introduced earlier in Arora et al.^20–23^. The framework utilizes a high-fidelity nonlinear and algebraic partial differential equation (NAPDE)-based simulation model, which is coupled with a constrained gray-box optimization solver for design, synthesis and optimization of PSA systems. Such an optimization-based analysis results in generating feasible process performance curves, which show the intricate relationship between several performance metrics of interest including product purity, recovery, production rate and BSF. To ensure PSA flexibility, we overdesign the process to provide higher oxygen purity at a nominal flow rate. Next, we fix the PSA design obtained and vary the PSA operation to maximize the map of the feasible operation defined by the area covered by the flow rate and the purity (Fig. 2). Therefore, when there arises a need for a higher flow rate or purity, the flexible PSA process can still meet the new product specification requirement with fixed design equipment without violating the purity constraint. Our analysis assumes that there is a surge column in which the product oxygen is collected, and the collected oxygen is then delivered at a constant flow rate. Consequently, we only require a single bed for constant oxygen delivery instead of the dual-bed setup.

Moreover, different zeolites can be used in conjunction with different PSA cycle configurations to develop air separation processes with varying performance and product specifications. Even though these adsorbents generally have a high working capacity and nitrogen/oxygen selectivity, there still exist significant variations in their adsorption isotherms, heats of adsorption, particle density and mass transfer resistances. These material-specific characteristics, when coupled with different cyclic configurations and operating conditions, can lead to significant differences in process performance. Therefore, to perform an extensive PSA flexibility analysis, we leverage the simulation-based optimization framework to evaluate the impact of varying material properties, cyclic configuration, bed design and operating conditions on process performance. The optimization studies are performed for candidate adsorbents including LiX, LiLSX and 5A zeolites in combination with both PSA- and PVSA-type cycles.

## Results

**Figure 3:**
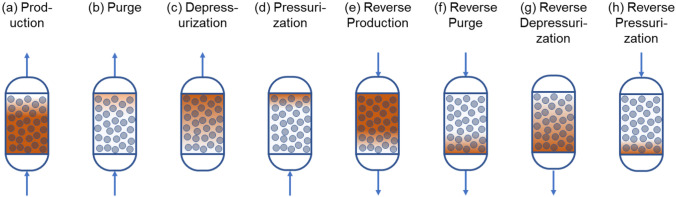
Graphical depiction of 8 process operation modes that constitute a PSA-based MOC cycle. The blue circles denote the adsorbent particles whereas the darker orange shade represents the part of the adsorbent column saturated with oxygen adsorbate.

With the development of adsorbents with high nitrogen/oxygen selectivity and efficient cyclic processes, adsorption-based technologies have undergone a significant reduction in capital and operating costs, and are competitive with cryogenic distillation^24^. Typically, the adsorption processes for air separation operate under a pressure swing, and are based on a Skarstrom-type cycle consisting of four different steps: (1) product generation at high pressure, (2) depressurization, (3) low-pressure purge and (4) pressurization. The individual operation modes that constitute a single-bed PSA cycle are shown in Fig. 3. In a multi-bed operation, additional cycle steps (e.g., pressure equalization) could also be included to improve overall process efficiency. During the first step, the less strongly adsorbed oxygen product is collected in the effluent stream, and the more strongly adsorbed nitrogen is captured by the adsorbent. Next, the adsorbent present inside the bed is regenerated with a combination of depressurization and purging steps.

In a PSA cycle, adsorbent is typically achieved through a sequence of steps which includes bed depressurization to a lower pressure, bed purging through a purge stream, and bed pressurization through a pressurization gas feed. In case of PSA-based air separation processes, a small fraction of the oxygen product produced during the initial production step of the cycle can be utilized as purge and pressurization gas feeds later during bed regeneration stages. Reusing the oxygen product later during purge and pressurization steps helps in regenerating the adsorbent column more efficiently, and in improving the oxygen product purity generated during the production step of the cycle^4^. As oxygen is produced only during a fraction of overall cycle operation, multiple beds can be designed and operated in an integrated fashion to result in continuous oxygen product generation. However, in this work, we focus on developing and analyzing single-bed PSA processes as they are less capitally expensive and result in a smaller unit operation size.

### Process simulation

Here, we solve the process simulation model for design and optimization of PSA- and PVSA-based MOCs with varying performance levels. The major focus is to evaluate different candidate adsorbents (i.e., LiX, LiLSX, 5A) and cycle operating conditions on the process performance metrics which include oxygen product purity and recovery, production rate and BSF. For given product specifications, a higher oxygen recovery and lower BSF improve the process economics. Depending on the application at hand, a specific set of operating conditions and adsorbent can be used to meet desired objectives.

**Figure 4:**
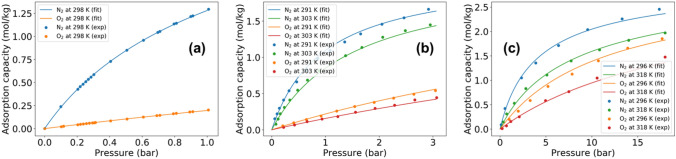
Experimental equilibrium adsorption capacity data and fitted isotherms for candidate adsorbents in adsorption-based MOC. (**a**) LiX, (**b**) LiLSX and (**c**) 5A zeolites with experimental data of Rege and Yang^25^, Zhu et al.^13^ and Talu et al.^26^, respectively.

For the candidate adsorbents considered, the equilibrium loading capacity of oxygen and nitrogen are obtained from experimental data in literature. Specifically, the equilibrium data for LiX, LiLSX and 5A zeolites are obtained from Rege and Yang^25^, Zhu et al.^13^ and Talu et al.^26^, respectively. A nonlinear programming formulation is then solved to fit the dual-site Langmuir adsorption isotherms on experimental data wherein the objective function is to minimize the mean squared error. Figure 4 shows the fitted adsorption isotherm curves and the experimental data for LiX, LiLSX and 5A zeolite adsorbents, and Table 2 reports the corresponding fitted isotherm parameters. In addition, Table 2 reports the heat of adsorption and the LDF mass transfer coefficient values for oxygen and nitrogen adsorption on these zeolites. The rest of the simulation parameters have been reported in the Supplementary Information.

**Table 2:** Fitted isotherm parameters, heat of adsorption, mass transfer coefficient, and particle characteristics for candidate adsorbent materials.

Parameter	Unit	LiX zeolite^13,25^		LiLSX zeolite^13,27^		5A zeolite^26,28^	
		$\hbox {O}_{2}$	$\hbox {N}_{2}$	$\hbox {O}_{2}$	$\hbox {N}_{2}$	$\hbox {O}_{2}$	$\hbox {N}_{2}$
$m_1$	mol kg$^{-1}$	1.5	1.5	1.5	1.5	1.5	1.5
$m_2$	mol kg$^{-1}$	0.6075	1.5	1.5	0.6613	1.5	1.5
$b_{o,1}$	Pa$^{-1}$	10$^{-10}$	10$^{-10}$	10$^{-10}$	10$^{-10}$	10$^{-10}$	10$^{-10}$
$b_{o,2}$	Pa$^{-1}$	10$^{-10}$	10$^{-10}$	10$^{-10}$	10$^{-10}$	10$^{-10}$	10$^{-10}$
$\Delta U_{1}$	J mol$^{-1}$	$-21774.6$	$-29195$	$-19606.9$	$-27923.9$	$-22312.1$	$-26140.8$
$\Delta U_{2}$	J mol$^{-1}$	$-24624.1$	$-26260.5$	$-22906.2$	-27923.9	$-22312.1$	$-23284.3$
$\Delta H$	J mol$^{-1}$	$-13221.44$	$-23430.4$	$-14661.44$	$-25451.65$	$-13221.44$	$-22886.48$
LDF constant	s$^{-1}$	–	–	104.07	42.84	12.07	4.02
Particle density	kg ads. m$^{-3}$ ads.	1200		1035		1160	
Heat capacity	J kg$^{-1}$ K$^{-1}$	1172		1172		1338.9	
Particle porosity	–	0.33		0.33		0.65	

Before performing the optimization case studies, several simulations are performed to generate process simulation data, visualize the process performance metrics, and obtain a reasonably good initial guess for optimization case studies. The MATLAB function lhsdesign is utilized for generating a set of space-filling input simulation points using latin hypercube sampling, which consists of varying air feed flow rate, step pressure levels and duration, purge flow rate and adsorbent packing density.

**Figure 5:**
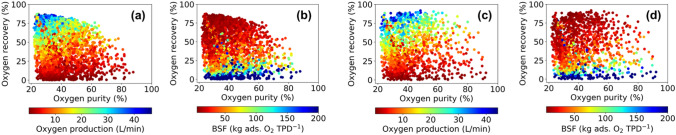
Process simulation data and output performance metrics for LiX zeolite. (**a**, **b**) PSA cycle and (**c**, **d**) PVSA cycle.

**Figure 6:**
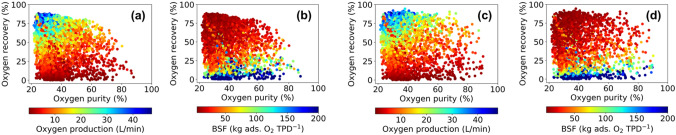
Process simulation data and output performance metrics for LiLSX zeolite. (**a**, **b**) PSA cycle and (**c**, **d**) PVSA cycle.

**Figure 7:**
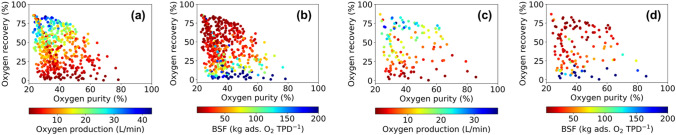
Process simulation data and output performance metrics for 5A zeolite. (**a**, **b**) PSA cycle and (**c**, **d**) PVSA cycle.

In the simulations performed, the feed composition of oxygen product used during purge and pressurization steps is prespecified. For instance, when the minimum oxygen purity requirement is 90%, a 90% pure oxygen feed is used for purging and pressurization. However, there may exist cases when there is a net consumption of oxygen due to longer purging times and/or sub-optimal process design and operation. In such cases, the net oxygen production amount is negative. For all the 3 adsorbents considered in this work, Figures 5, 6, 7 only show the simulation data wherein there is a net production of oxygen product, i.e., positive oxygen production amount. It should be particularly noted that 90% pure oxygen is utilized as purge and pressurization feed, and varying this feed composition will alter the results shown here.

In particular, Figs. 5, 6, 7 report the process performance metrics, i.e., oxygen purity, recovery, production rate and BSF, for the LiX, LiLSX and 5A zeolite adsorbents. In all these cases, there is a consistent tradeoff that is observed between oxygen purity and recovery, and increasing the desired oxygen product purity results in lower oxygen recovery values. A Pareto frontier can also be constructed using the simulated data wherein any increase in oxygen purity has associated tradeoff in terms of reduction in oxygen recovery. For instance, in the case of LiX and PSA cycle operation, the oxygen recovery drops from 87.3 to 24.7% when the oxygen purity obtained increases from 24.7 to 87.8% (Fig. 5a). Moreover, the performance of PVSA cycle is better compared to PSA cycle due to more effective adsorbent regeneration. This pattern is also consistent across all the materials, as both maximum oxygen recovery and oxygen purity obtained is higher for PVSA cycles. For example, the maximum oxygen purity obtained for PVSA-based oxygen concentrator is 92.1%, 90.2% and 85.7% for LiX, LiLSX and 5A, respectively, which is higher than purity levels for PSA operation, i.e., 87.8%, 88% and 78.2%.

In addition, Figs. 5, 6, 7 also highlight the effect of varying purity-recovery levels on oxygen production rate and BSF. When oxygen purity values are closer to air composition (i.e., 21% oxygen), higher oxygen product rates could be obtained as there is not much air separation required, and a majority of incoming air can be recovered as a low-purity product. However, when the oxygen product has higher purity levels, a significant amount of PSA/PVSA cycle time is spent on bed regeneration to facilitate efficient air separation thereby leading to lower oxygen production amounts. As an example, oxygen production rate drops from 47.2 to 0.5 L/min when oxygen purity increases from 28.2 to 88%, respectively, for PSA-based air separation using LiLSX adsorbent (Fig. 6a). Due to a similar reason, BSF also decreases with increasing oxygen purity values in the observed Pareto frontiers. For lower oxygen purity, the adsorbents have higher oxygen productivity (i.e., amount of oxygen produced per unit mass of adsorbent) as the adsorbents need not be regenerated frequently.

Compared to LiX and LiLSX adsorbents, 5A zeolite has substandard process performance, especially in terms of oxygen purity obtained, due to lower nitrogen/oxygen selectivity and equilibrium adsorption capacity. Despite using 90% pure oxygen for purge and pressurization steps, the process is unable to meet the oxygen purity requirements with maximum purity observed only being 78.2% and 85.7% for PSA and PVSA, respectively. In addition, Fig. 7 shows conservative estimates of oxygen purity obtained using 5A with 90% pure oxygen purge and pressurization, and further simulations with low-purity purge and pressurization streams indicated that 5A-based adsorption process is unable to produce high-purity oxygen. Consequently, we do not consider 5A as a prospective candidate adsorbent in forthcoming optimization case studies.

**Table 3:** Initial guess for optimization case studies.

Parameter/output metric	Unit	LiX		LiLSX	
		PSA	PVSA	PSA	PVSA
$\hbox {O}_{2}$ Purity	%	80.85	89.96	81.94	87.97
$\hbox {O}_{2}$ Recovery	%	40.42	57.69	47.85	60.7
$\hbox {O}_{2}$ Production	L min$^{-1}$	8.12	9.9	15.99	20.3
Bed size factor	kg ads. $\hbox {O}_{2}$ TPD$^{-1}$	42.88	30.79	20.08	15.83
Cycle configuration	–	(1) Production	(1) Production	(1) Production	(1) Production
		(2) Reverse depres.	(2) Reverse depres.	(2) Depressurization	(2) Depressurization
		(3) Reverse purge	(3) Reverse purge	(3) Reverse purge	(3) reverse Purge
		(4) Pressurization	(4) Pressurization	(4) Pressurization	(4) Pressurization
$P_1$	bar	3.4	3.5	3.5	3.5
$P_2$	bar	1	0.5	1	0.54
$P_3$	bar	1	0.5	1	0.54
$t_1$	s	3.8	6	3	3
$t_2$	s	1	1	1	1
$t_3$	s	4.6	6.1	1	1
$t_4$	s	1	1	1	1
$\dot{n}_1$	mol s$^{-1}$	0.2	0.14	0.24	0.24
Purge flow velocity factor	–	0.42	0.73	2.22	2.22
$\rho _{b,ads}$	kg ads. m$^{-3}$ bed	718.03	633.43	662.78	662.78

The process simulation data generated here is used to obtain initial guesses for the optimization case studies. While selecting a specific input simulation point as an initial guess, it is ensured that the corresponding output oxygen purity is high with a moderately high value of oxygen recovery. This ensures that the initial guess selected can meet the desired oxygen purity constraint relatively easily, and could also result in high oxygen recovery objective values. Table 3 reports the initial guess for both PSA and PVSA cycles for LiX and LiLSX adsorbents. It can be observed that for all of the initial guess points, the high and low step pressure values are closer to their respective bounds, and the 4-step cycle configuration consists of production, reverse depressurization, reverse purge and pressurization steps.

### Optimization case studies

#### Oxygen recovery maximization

A higher oxygen recovery leads to lower compression costs as a majority of incoming oxygen is captured in the product stream, thereby reducing the amount of air feed compressed. Therefore, we maximize the oxygen recovery to obtain optimal cycle pressure and duration, air and purge feed specifications and adsorbent packing density subject to varying levels of minimum oxygen purity (50, 55, 60, …, 95%) and production rates (1, 5, 10 and 15 L/min). In addition, the optimization is performed for both PSA and PVSA cycles for LiX and LiLSX adsorbents. Overall, this results in a set of 160 optimization runs.

**Figure 8:**
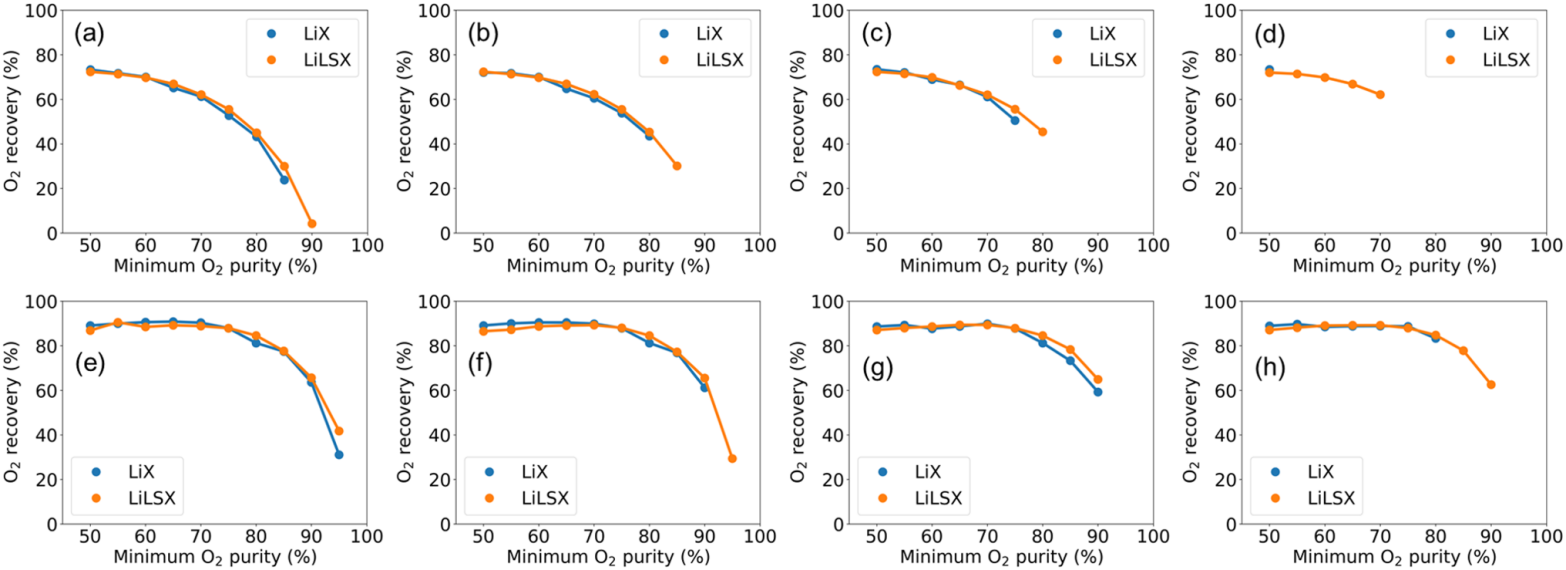
Purity-recovery Pareto frontier obtained using optimization-based analysis. (**a**–**d**) PSA with minimum production (**a**) 1, (**b**) 5, (**c**) 10 and (**d**) 15 L/min. (**e**–**h**) PVSA with minimum production (**e**) 1, (**f**) 5, (**g**) 10 and (**h**) 15 L/min.

Figure 8 shows the purity-recovery tradeoff curves for both LiX and LiLSX adsorbents with varying minimum oxygen purity and production and cycle types. The figure illustrates a strong inverse relationship between oxygen recovery and purity. Therefore, producing high-purity oxygen would require higher compression costs as a significant amount of incoming oxygen is lost in a PSA/PVSA cycle. For instance, to produce at least 1 L/min of 85% pure oxygen using PSA cycle and LiX adsorbent, only 23.8% oxygen recovery is obtained (Fig. 8a). However, the oxygen recovery could be significantly improved by using a PVSA cycle operation wherein up to 77.4% recovery is observed (Fig. 8e). In addition, PVSA processes also outperform PSA processes in terms of obtaining a feasible solution with higher minimum oxygen purity levels. This improvement in purity-recovery Pareto frontiers is consistent across all the optimization results wherein a significant improvement in process performance is observed due to bed regeneration at sub-atmospheric pressure. However, such an improvement comes at the cost of installing a vacuum pump, which reduces the overall compactness and modularity of an oxygen concentrator unit.

With increasing minimum oxygen purity and production amount, several optimization runs are unable to return a feasible solution. Figure 8 shows this trend wherein for many of the cases, higher oxygen purity levels do not report a feasible solution. This indicates the performance limits of adsorption-based air separation for producing different product specifications using LiX and LiLSX adsorbents. In addition, for all the optimization runs, LiLSX generally performs better than LiX in terms of both oxygen purity levels and production rates obtained. Even though the maximum recovery levels are similar for both adsorbents, LiLSX-based oxygen concentrator units can satisfy more stringent oxygen purity and/or product rate constraints. To illustrate, for a PSA process with a minimum oxygen production rate of 15 L/min, LiLSX can produce 50–70% pure oxygen product, whereas LiX is unable to produce oxygen with more than 50% purity (Fig. 8d). A similar trend is also observed for PVSA-based air separation with a minimum production rate of 15 L/min (Fig. 8h).

Table 4 summarizes the optimization results, and the corresponding optimal decision variables, for varying levels of minimum oxygen purity and production rate. Specifically, the results are reported for the oxygen production rate in the range 5–10 L/min and an oxygen purity in the range 70–95% as these ranges are within typical product specifications for medical use. For all the cases, the PSA/PVSA cycle configurations consist of the following 4 steps: (1) production, (2) reverse depressurization, (3) reverse purge and (4) pressurization. It must be noted that the objective function consisted of maximizing oxygen recovery. To be comprehensive, Table 4 reports all of the process performance metrics including BSF, which is not a part of objective function or constraints. For a few cases, the oxygen purity reported is less than the corresponding minimum oxygen threshold due to the constraint violation tolerance included in the optimization case studies.

In the analysis presented here, we fixed the length and radius of the adsorption column to be 0.127 and 0.05 m, respectively. These bed geometry parameters, along with the rest of the simulation parameters, are reported in the Supplementary Information. The adsorption bed dimensions have been taken such that the whole unit is modular and compact, and are based on typical dimensions reported in the literature for portable MOCs^7,12^. These bed dimensions, along with the LiLSX-based PVSA cycles optimized in Table 4, can be utilized for developing a compact MOC that can deliver 95% pure oxygen at 5 L/min and 90% pure oxygen at 10 L/min. It should however be noted that developing a PVSA-based MOC would require installing two separate pumps for compression and vacuum. To make the MOC more compact, only PSA-based cycle designs can also be utilized although it results in lower oxygen purity and production rate. For instance, in case of LiLSX adsorbent, 85% pure oxygen can be obtained a flowrate of 5 L/min using PSA cycle design.

**Table 4:** Optimization results for PSA and PVSA cycles using LiX and LiLSX zeolites with maximum oxygen recovery, and varying minimum oxygen purity and production levels.

Parameter/output metric	Unit	$PC_{{\mathrm{O}}_{2}}$ $\ge$ 5 L/min			$PC_{{\mathrm{O}}_{2}}$ $\ge$ 10 L/min		
		$P_{{\mathrm{O}}_{2}}$ $\ge$ 70%	$P_{{\mathrm{O}}_{2}}$ $\ge$ 75%	$P_{{\mathrm{O}}_{2}}$ $\ge$ 80%	$P_{{\mathrm{O}}_{2}}$ $\ge$ 70%	$P_{{\mathrm{O}}_{2}}$ $\ge$ 75%	$P_{{\mathrm{O}}_{2}}$ $\ge$ 80%
**PSA cycle with LiX adsorbent**							
$\hbox {O}_{2}$ purity	%	69.96	74.99	79.93	69.98	75.09	-
$\hbox {O}_{2}$ recovery	%	60.40	53.81	43.46	61.05	50.54	-
$\hbox {O}_{2}$ production	L min$^{-1}$	10.92	16.54	9.58	17.11	10.54	-
Bed size factor	kg ads. $\hbox {O}_{2}$ TPD$^{-1}$	33.24	22.87	39.49	22.11	32.24	–
$P_1$	bar	3.5	3.5	3.5	3.5	3.5	–
$P_2$	bar	1	1	1	1	1	–
$P_3$	bar	1	1	1	1	1	–
$t_1$	s	4.7	3.8	4.7	4.2	3.8	–
$t_2$	s	1	1	1	1	1	–
$t_3$	s	5.9	1.9	4.5	2.1	4.5	–
$t_4$	s	1	1	1	1	1	–
$\dot{n}_1$	mol s$^{-1}$	0.17	0.22	0.19	0.2	0.2	–
Purge flow velocity factor	-	0.46	1.34	0.78	1.19	0.54	–
$\rho _{b,ads}$	kg ads. m$^{-3}$ bed	748.6	780	780	780	700.66	–

Parameter/output metric	Unit	$PC_{{\mathrm{O}}_{2}}$ $\ge$ 5 L/min			$PC_{{\mathrm{O}}_{2}}$ $\ge$ 10 L/min		
		$P_{{\mathrm{O}}_{2}}$ $\ge$ 80%	$P_{{\mathrm{O}}_{2}}$ $\ge$ 85%	$P_{{\mathrm{O}}_{2}}$ $\ge$ 90%	$P_{{\mathrm{O}}_{2}}$ $\ge$ 80%	$P_{{\mathrm{O}}_{2}}$ $\ge$ 85%	$P_{{\mathrm{O}}_{2}}$ $\ge$ 90%
**PVSA cycle with LiX adsorbent**							
$\hbox {O}_{2}$ purity	%	79.9	84.99	90.03	79.7	84.93	90.03
$\hbox {O}_{2}$ recovery	%	81.23	76.8	61.18	81.21	73.25	59.24
$\hbox {O}_{2}$ production	L min$^{-1}$	14.12	14.25	11.99	13.92	12.31	10.62
Bed size factor	kg ads. $\hbox {O}_{2}$ TPD$^{-1}$	21.56	25.42	27.7	21.46	23.89	28.76
$P_1$	bar	3.49	3.5	3.5	3.5	3.5	3.5
$P_2$	bar	0.5	0.5	0.5	0.5	0.5	0.5
$P_3$	bar	0.5	0.5	0.5	0.5	0.5	0.5
$t_1$	s	6.2	6.6	6.2	6.1	5	6.2
$t_2$	s	1	1	1	1	1	1
$t_3$	s	5.9	6.8	5.7	5.9	7.4	5.9
$t_4$	s	1	1	1	1	1	1
$\dot{n}_1$	mol s$^{-1}$	0.14	0.15	0.16	0.14	0.17	0.14
Purge flow velocity factor	–	0.53	0.6	0.9	0.49	0.43	0.86
$\rho _{b,ads}$	kg ads. m$^{-3}$ bed	627.72	747.02	684.89	616.06	606.55	629.81

Parameter/output metric	Unit	$PC_{{\mathrm{O}}_{2}}$ $\ge$ 5 L/min			$PC_{{\mathrm{O}}_{2}}$ $\ge$ 10 L/min		
		$P_{{\mathrm{O}}_{2}}$ $\ge$ 75%	$P_{{\mathrm{O}}_{2}}$ $\ge$ 80%	$P_{{\mathrm{O}}_{2}}$ $\ge$ 85%	$P_{{\mathrm{O}}_{2}}$ $\ge$ 75%	$P_{{\mathrm{O}}_{2}}$ $\ge$ 80%	$P_{{\mathrm{O}}_{2}}$ $\ge$ 85%
**PSA cycle with LiLSX adsorbent**							
$\hbox {O}_{2}$ purity	%	74.89	79.99	84.72	74.94	80	–
$\hbox {O}_{2}$ recovery	%	55.46	45.32	30.14	55.56	45.45	–
$\hbox {O}_{2}$ production	L min$^{-1}$	16.9	14.53	9.02	14.69	12.35	–
Bed size factor	kg ads. $\hbox {O}_{2}$ TPD$^{-1}$	19.27	22.44	36.15	22.2	26.42	–
$P_1$	bar	3.49	3.5	3.49	3.49	3.5	–
$P_2$	bar	1	1	1	1	1	–
$P_3$	bar	1	1	1	1	1	–
$t_1$	s	2.7	2.5	3	3.6	3.4	–
$t_2$	s	1	1	1	1	1	–
$t_3$	s	1	1	1.4	1	1	–
$t_4$	s	1	1	1	1	1	–
$\dot{n}_1$	mol s$^{-1}$	0.23	0.25	0.23	0.17	0.18	–
Purge flow velocity factor	–	1.83	1.9	1.97	2.44	2.5	–
$\rho _{b,ads}$	kg ads. m$^{-3}$ bed	671.57	672.6	672.75	672.38	672.75	–

Parameter/output metric	Unit	$PC_{{\mathrm{O}}_{2}}$ $\ge$ 5 L/min			$PC_{{\mathrm{O}}_{2}}$ $\ge$ 10 L/min		
		$P_{{\mathrm{O}}_{2}}$ $\ge$ 85%	$P_{{\mathrm{O}}_{2}}$ $\ge$ 90%	$P_{{\mathrm{O}}_{2}}$ $\ge$ 95%	$P_{{\mathrm{O}}_{2}}$ $\ge$ 85%	$P_{{\mathrm{O}}_{2}}$ $\ge$ 90%	$P_{{\mathrm{O}}_{2}}$ $\ge$ 95%
**PVSA cycle with LiLSX adsorbent**							
$\hbox {O}_{2}$ purity	%	85.05	89.97	95	85	90	–
$\hbox {O}_{2}$ recovery	%	77.22	65.51	29.37	78.32	64.89	–
$\hbox {O}_{2}$ production	L min$^{-1}$	26.8	22.53	9.67	27.5	21.67	–
Bed size factor	kg ads. $\hbox {O}_{2}$ TPD$^{-1}$	12.15	14.48	30.07	11.86	15.05	–
$P_1$	bar	3.5	3.5	3.5	3.5	3.5	–
$P_2$	bar	0.5	0.5	0.5	0.5	0.5	–
$P_3$	bar	0.5	0.5	0.5	0.5	0.5	–
$t_1$	s	3	3.2	2.8	3.2	3.2	–
$t_2$	s	1	1	1	1	1	–
$t_3$	s	1.1	1.3	1.2	1	1.4	–
$t_4$	s	1	1	1	1	1	–
$\dot{n}_1$	mol s$^{-1}$	0.25	0.25	0.25	0.24	0.24	–
Purge flow velocity factor	–	1.87	2.32	2.65	2.45	2.05	–
$\rho _{b,ads}$	kg ads. m$^{-3}$ bed	671.26	672.75	599.69	672.75	672.75	–

#### Bed size factor minimization

Here, we minimize the bed size factor to result in more compact and productive oxygen concentrator units. Therefore, in the optimization runs, the objective function is modified which now consists of minimizing BSF value while meeting minimum oxygen production rate and purity levels. Similar to the previous case, we perform the optimization runs for a range of minimum production rates (1, 5, 10 and 15 L/min) and minimum purity (50, 55, 60, …, 95%) to analyze the dependence of product specifications on process performance. In addition, the analysis has been performed for both LiX and LiLSX adsorbents, and PSA and PVSA cycles, thereby leading to a set of 160 optimization runs in total.

**Figure 9:**
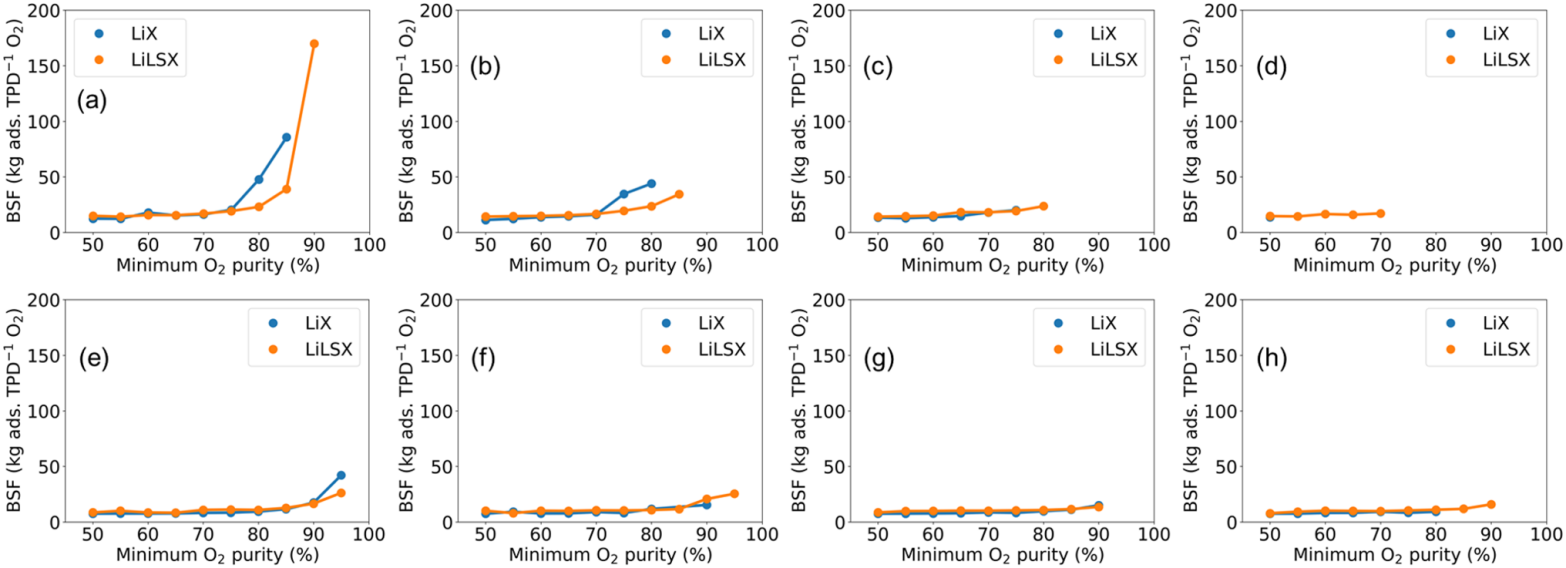
Purity-BSF Pareto frontier obtained using optimization-based analysis. (**a**–**d**) PSA with minimum production (**a**) 1, (**b**) 5, (**c**) 10 and (**d**) 15 L/min. (**e**–**h**) PVSA with minimum production (**e**) 1, (**f**) 5, (**g**) 10 and (**h**) 15 L/min.

Figure 9 shows the variation of BSF with varying minimum oxygen purity levels for different adsorbent materials, cycle operation and minimum oxygen production rate. Typically, we observe that the BSF value increases with an increase in minimum oxygen purity level. For instance, in the case of PSA cycle operation with a minimum 1 L/min oxygen production rate, BSF increases from 14.2 to 169.9 when the minimum oxygen purity threshold increases from 50 to 90% for LiLSX zeolite (Fig. 9a). This observation is attributed to the fact that the adsorbent needs to be regenerated more frequently to obtain a higher product purity. The net average production rate of oxygen decreases per cycle as a large fraction of cycle time is spent on bed regeneration. In addition, when breakthrough occurs, the production step needs to be cut off earlier to obtain a high purity product. Due to these factors, higher BSF values are obtained for higher oxygen purity product. Consequently, the size of the adsorbent bed required would also increase as more adsorbent needs to be packed for generating high-purity product.

Similar to the previous optimization case study for oxygen recovery maximization, LiLSX consistently performs better than LiX in terms of lower BSF values, and higher oxygen purity and production rates. To illustrate, for producing at least 1 L/min oxygen with 85% purity using PSA cycle, a BSF of 39.1 and 85.6 is obtained using LiLSX and LiX adsorbents, respectively (Fig. 9a). In addition, LiLSX outperforms LiX in terms of obtaining feasible solutions for high purity constraints. For example, in Fig. 9d with at least 15 L/min oxygen production rate, LiLSX can produce up to 70% pure oxygen whereas LiX could only match up to 50% product purity.

### Mapping flexible PSA operation to product specifications

Previously, we optimized both design and operational variables for oxygen production using PSA and PVSA processes. However, in this section, the objective is to fix the design variables of the process and map the effect of varying operating conditions on product specifications. As the two major product specifications are oxygen purity and production rate, we perform different optimization runs for maximizing oxygen purity with varying levels of minimum production rate. To reduce the computational expense of obtaining an optimal fixed design, we utilize the previous solution obtained with the highest oxygen purity. More specifically, for PSA system, we fix the design variable (i.e., adsorbent packing density) to be the same as in the solution vector that resulted in 80% and 75% purity for LiLSX and LiX, respectively (Fig. 8c). Similarly, in the case of PVSA, the design solution that resulted in 90% and 80% oxygen is utilized (Fig. 8h). This results in an overdesigned PSA/PVSA process that is capable of meeting high-purity requirements, and if required, a higher production rate can also be obtained with a small decrease in purity. Next, we perform optimization runs to maximize the feasible product specification area, which also helps us in obtaining the product specification Pareto curve.

**Figure 10:**
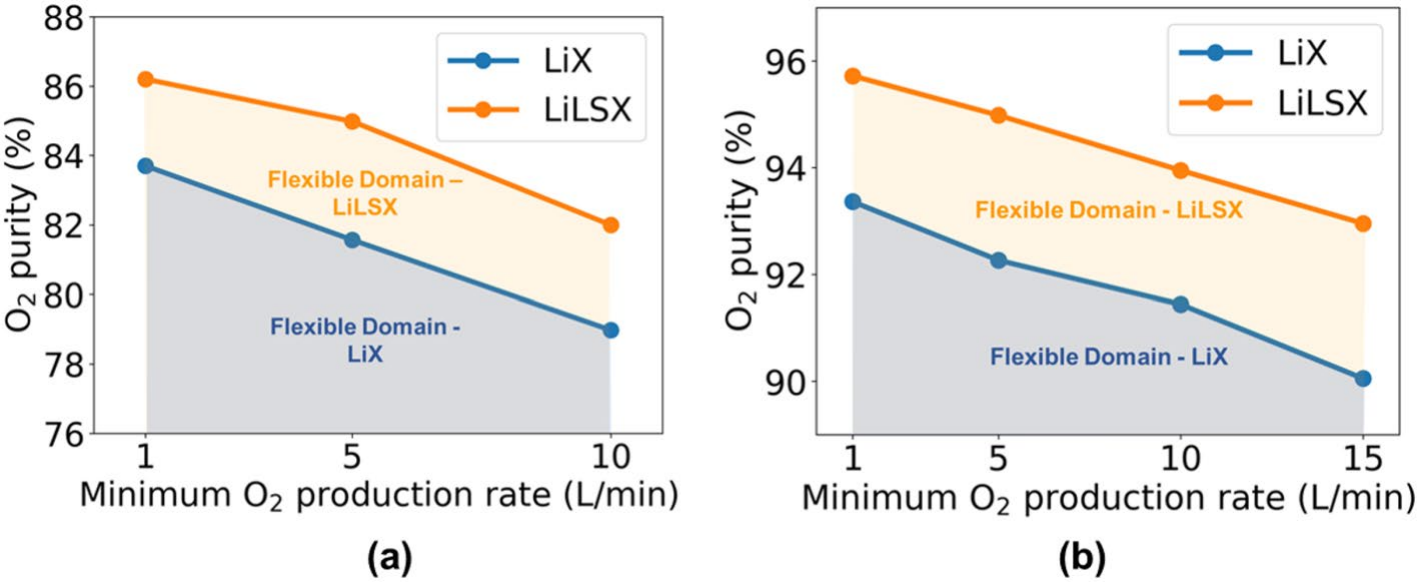
Flexible (**a**) PSA and (**b**) PVSA operation for generating product oxygen with different purity and production rate specifications.

The tradeoff curve between oxygen purity and production rate for LiX and LiLSX zeolites can be found in Figure 10. The figure also shows the variation in purity-production tradeoff curve for both PSA and PVSA cycle operations. Similar to earlier results, LiLSX performs better than LiX, and is capable of meeting higher purity and/or higher production rate requirements. Using LiLSX, an oxygen purity in the range 82–86.2% and 93–95.7% could be obtained, respectively, for PSA and PVSA systems. In the case of LiX, a lower oxygen purity of 79–83.7% and 90–93.4%. is observed. Any product specification combination of product purity and production rate that lies to the bottom-left of tradeoff curve is feasible. This is because the product oxygen purity could always be reduced by mixing with fresh air, and the inlet air feed flow rate could be adjusted to meet desired product demand. However, any product specification to the top-right of tradeoff curve is unattainable due to performance limitations of the PSA and PVSA systems.

## Discussion

We investigated portable medical oxygen concentrators (MOCs) for delivering medical grade oxygen to patients suffering from severe lung conditions including COVID-19 and COPD. More specifically, we designed and optimized adsorption-based flexible MOC systems that are capable of producing oxygen with time-varying flow rate and purity requirements. The flexible design and operation of MOC units are inherently advantageous as the same MOC unit can be reconfigured to satisfy varying oxygen requirements. In this work, the specific adsorption technologies considered for MOC include pressure swing adsorption (PSA) and pressure vacuum swing adsorption (PVSA), which are dynamically operated with multiple operating modes and periodic cycles. To handle the associated challenges with optimal PSA/PVSA design and operation, we employ a simulation-based optimization framework for optimal synthesis, design and operation of adsorption-based MOC systems.

The results strongly indicate that LiLSX adsorbent performs significantly better than both LiX and 5A adsorbents. Specifically, using a PVSA cycle, LiLSX-based adsorption process produces 90% pure oxygen at a flow rate of 21.7 L/min with a high oxygen recovery of 64.9%. Moreover, using LiLSX, a high oxygen purity of 95% can be produced at a flow rate of 9.7 L/min. In the subsequent flexibility analysis, we determined the feasible domain of operation for PSA and PVSA systems. Using LiLSX-based PVSA operation, the flexible operation yielded oxygen product of different specifications with the purity and flow rate limits in the range 93–95.7% and 1–15 L/min, respectively.

We envision the use of such a flexible PSA- and PVSA-based MOC in a cyber-physical system (CPS) wherein the patients are constantly monitored for blood oxygen level configuration, and a controller determines the optimal control action policies to reconfigure the MOC operation to meet patient’s oxygen requirement in real-time. Even though the current analysis has focused primarily on optimizing flexible MOC process performance and determining the feasible domain of output product specifications, the results can be readily extended to implement the entire CPS workflow. To achieve this vision, the additional step required consists of training a process controller offline using optimal action policies. Consequently, the controller can automatically reconfigure MOC operation in real-time to meet varying product specifications.

**Figure 11:**
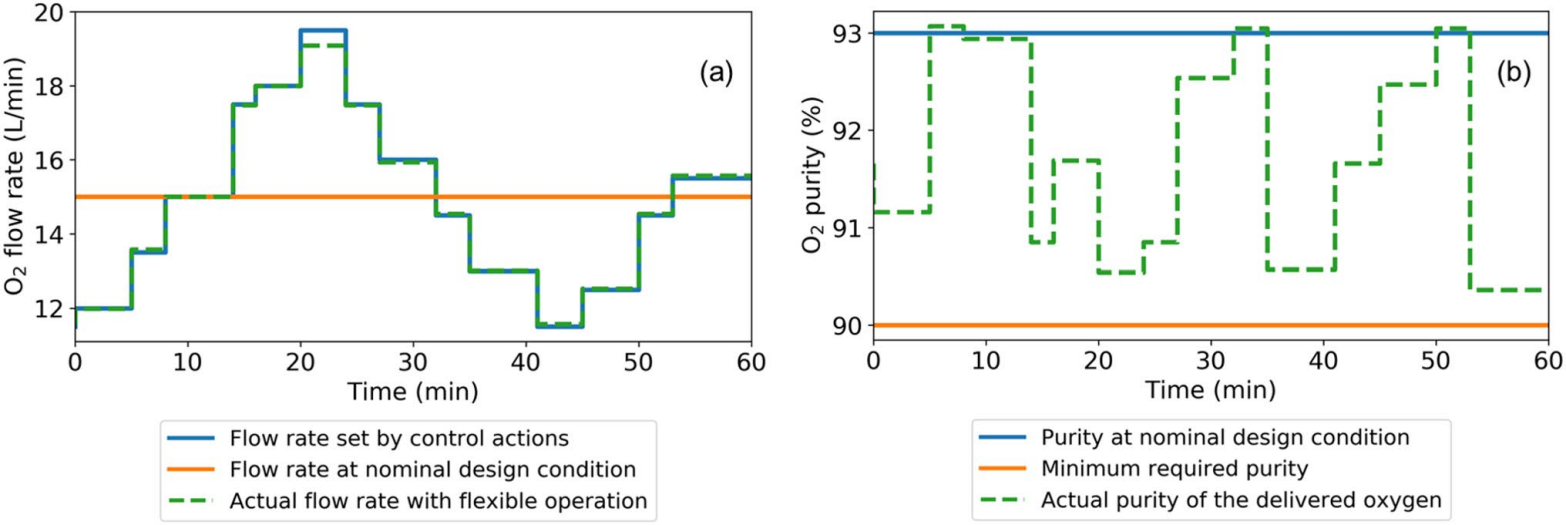
Comparison between actual and target oxygen (**a**) flow rate and (**b**) purity for meeting time-varying oxygen flow rate demand with 90% minimum purity.

To illustrate, we extended our optimization analysis to showcase how the variations in required oxygen flowrate and purity can be met with the use of flexible MOCs (Fig. 11). Even though the MOC has been designed for nominal flow rate and purity levels, appropriate operation changes can be made by the controller to attain the oxygen specification requirements of a patient. Figure 11 shows the wide variations in flow rate that can be obtained while maintaining at least 90% product purity. It should be particularly noted that this result has been obtained by performing several rigorous optimizations. However, achieving real-time oxygen demand delivery necessitates the need to develop a high fidelity controller with quick response time.

## Methods

### First principles-based adsorption model

The first principles-based mathematical model describing the adsorption physics has been adopted from Arora et al.^20^, and is based on a 1-dimensional, non-isothermal, non-isobaric and non-adiabatic model. The model computes temperature, concentration and pressure gradients along the axial column length and time dimension, and neglects any variations in the state variables along the radial direction. Consequently, the model consists of a set of nonlinear algebraic and partial differential equations (NAPDE). The overall conservation equations used in the model have been described in the Supplementary Information.

To reduce computational expense, the gas and solid phases are not modeled separately, and a pseudo-homogeneous adsorber model is assumed with negligible intra-particle concentration and temperature gradients. The steady-state momentum balance (Darcy’s law) and linear driving force (LDF) equation are used to describe the pressure drop and adsorption kinetics in the model. For obtaining the equilibrium adsorption capacity of different adsorbate species on adsorbent, experimental data is fitted using the dual-site Langmuir adsorption isotherms of the following form:

1a $$\begin{aligned} q^{*}_{i} = \sum _{s} \dfrac{m_{i,s} b_{i,s} P_i}{1 + b_{i,s} P_i}, \end{aligned}$$

where $s \in \{1,2\}$ are the two adsorption sites, *i* represents the chemical species, $m_{i,s}$ is the solid phase adsorption capacity for site s, and $P_i$ is the partial pressure. In the above equation, $b_{i,s}$ is calculated as follows:

1b $$\begin{aligned} b_{i,s} = b_{o,i,s} \exp \left( \dfrac{ -\Delta U_{i,s}}{RT} \right) , \end{aligned}$$

where, $b_{o,i,s}$ and $\Delta U_{i,s}$ are the isotherm fitting parameters.

### Process simulation

For solving the NAPDE model, the finite volume method is utilized to discretize the partial differential equations into an equivalent ordinary differential equation (ODE)-based model, which is then solved using ode15s and ode23s solvers in MATLAB. The interested reader is referred to Arora et al^20^ to learn more about the discretization scheme and solution strategy used for solving the NAPDE model.

As the simulation-based optimization solver obtains optimal PSA cyclic configuration and operation, additional assumptions are imposed to result in a computationally tractable problem. These assumptions are as follows: (1) inlet air feed consists of 21% oxygen and 79% nitrogen, and is assumed to be free of water and argon^3,29^; (2) purge and pressurization feed consist of a pre-specified composition of oxygen and nitrogen; (3) purge and pressurization feed consists of a pre-specified composition of oxygen and nitrogen; (4) for PSA cycles, the lowest attainable pressure is 1 bar, and for PVSA cycles, the lowest pressure is 0.5 bar; (5) the maximum number of PSA cycle steps is 4; (6) 50 cycles are simulated for attaining cyclic steady state as output metrics are observed to converge for these many cycles; (7) initially, the bed is saturated with air at first step pressure and feed temperature of a PSA cycle; and (8) the first step of a PSA cycle is the oxygen production step wherein air is fed to the column.

### Process optimization

The PSA process model is optimized using the two-phase algorithm by Bajaj et al^30^, and more details regarding the simulation-based optimization model can be found elsewhere^21^. For PSA-based MOC unit optimization, the objective function is either the maximization of oxygen recovery or minimization of BSF. Optimizing these metrics leads to a PSA process with low compression costs, more efficient use of adsorbent, and compact and modular PSA units. As the end-use amount requirement of oxygen product differs, numerous optimization case studies are performed with varying levels of product specifications. In the overall optimization problem, there are 10 decision variables that are optimized to result in optimal cycle design and operation of PSA for air separation. These include air feed flow rate, 3 operating pressure variables, 4 cycle step durations, purge flow velocity factor and adsorbent bulk density.

Even though it is preferable to have a high adsorbent bulk density to adsorb as much nitrogen adsorbate as possible, it leads to higher feed interstitial velocity that reduces the bed residence time of incoming ambient air. In addition, a higher adsorbent bulk density leads to higher pressure drop which could lead to an undesirable drop in pressure of product outlet feed. Consequently, a higher adsorbent bulk density can lead to under-utilization of packed adsorbent and reduction in separation efficiency of the adsorbent. Therefore, it becomes crucial to balance this tradeoff to determine the optimal adsorbent bulk density.

The bounds on these decision variables can be found in the Supplementary Information. The air feed flow rate during the production step of a PSA cycle is varied between 0.01 and 0.25 mol/s. Such a variation in air flow rate was found to be sufficient to produce varying levels of oxygen production amounts. The pressure of each of PSA cyclic steps is varied between − 3.5 and 3.5 bar. It should be noted that the negative pressure signs are an abstract concept, introduced earlier in Arora et al.^21^, that help us in simultaneously optimizing both operating pressure and cycle design of PSA systems. To result in shorter PSA cycles, the step duration can vary between 1 and 10 s. Additional constraints are imposed in the overall optimization model to ensure that the duration of pressurization steps (i.e., pressurization and depressurization) hit the lower bound of 1 s, thereby resulting in a majority of cycle time spent during production and/or purging steps. The purge feed velocity is obtained by multiplying a factor with the air feed velocity during step 1, which can vary between 0.1 and 3. This results in a sufficiently flexible range for purging operation. The adsorbent bulk density is assumed to vary between 35 and 65% of adsorbent particle density. This ensures that bulk packing densities are within realistic bounds with sufficient bed void fraction, thereby preventing high interstitial velocities which can lead to numerical instabilities.

## Supplementary Information

Supplementary Information 1.
