# Supplementary material for: Flexible oxygen concentrators for medical applications
Source: Sci Rep. 2021 Jul 12;11:14317. doi: 10.1038/s41598-021-93796-3 (PMC8275632; doi:10.1038/s41598-021-93796-3)
Supplement: Supplementary file 1 — Supplementary Information 1. [file 41598_2021_93796_MOESM1_ESM.pdf]

Supplementary Information for Flexible Oxygen Concentrators for Medical Applications

Akhil Arora, M. M. Faruque Hasan
Artie McFerrin Department of Chemical Engineering, Texas A&M University, College Station, TX 77843-3122, USA.

High-Fidelity Adsorption Simulation Model

The high-fidelity model used for simulating pressure swing adsorption (PSA) processes consists of a 1-dimensional, pseudo-homogeneous, non-isothermal, non-adiabatic and non-isobaric model. A set of nonlinear and algebaric partial differential equations (NAPDE) is used for describing the variation of species concentration, temperature and pressure along the bed length and time dimension. The first principles equations leveraged in the model are as follows.

The following equation represents the mass conservation of each chemical species i in the gas phase:

$$\varepsilon_t \frac{\partial C_i}{\partial t} = \varepsilon_b \frac{\partial}{\partial z}\left(D_L C \frac{\partial y_i}{\partial z}\right) - \varepsilon_b \frac{\partial (vC_i)}{\partial z} - \rho_{b,ads} \frac{\partial \overline{q}_i}{\partial t}. \tag{S1a}$$

Here, C_i and C are respectively the concentration of component i and total concentration in the gas phase, ε_b and ε_t are bed and total void fraction, y_i is component i gas phase mole fraction, $\rho_{b,ads}$ is the adsorbent bulk packing density. Moreover, v is the interstitial velocity, $\overline{q}_i$ is the component i solid phase concentration, D_L is the axial dispersion coefficient, and z and t are the space and time dimensions, respectively.

In the above component mass balance equation, the ideal gas law is applied to convert the concentration in terms of gas phase mole fraction, pressure and temperature. Consequently, the following equation is obtained:

$$\frac{\partial y_i}{\partial t} + \frac{y_i}{P}\frac{\partial P}{\partial t} - \frac{y_i}{T}\frac{\partial T}{\partial t} = D_L \frac{\varepsilon_b}{\varepsilon_t}\frac{T}{P}\frac{\partial}{\partial z}\left(\frac{P}{T}\frac{\partial y_i}{\partial z}\right) - \frac{\varepsilon_b}{\varepsilon_t}\frac{T}{P}\frac{\partial}{\partial z}\left(\frac{y_i v P}{T}\right) - \frac{\rho_{b,ads} RT}{\varepsilon_t P}\frac{\partial \overline{q}_i}{\partial t}, \tag{S1b}$$

where, y_i is the component i gas phase mole fraction, P is the gas phase pressure, and T is the gas phase temperature.

Eq. S1b represents the component mass balance for each chemical species i. To obtain the total mass balance equation, we sum over Eq. S1b for all component species $i \in I$. The resulting total mass balance expression is as follows:

$$\frac{\partial P}{\partial t} = \frac{P}{T}\frac{\partial T}{\partial t} - \frac{\varepsilon_b T}{\varepsilon_t}\frac{\partial}{\partial z}\left(\frac{Pv}{T}\right) - \frac{\rho_{b,ads} RT}{\varepsilon_t}\sum_{i\in I}\frac{\partial \overline{q}_i}{\partial t}. \tag{S1c}$$

For computing the temperature variations due to adsorption and adsorbent-column-wall interactions, the following heat balance equation is utilized:

$$\begin{aligned}\left(\rho_{b,ads} C_{p,ads} + C_{p,a}\rho_{b,ads}\sum_{i\in I}\overline{q}_i\right)\frac{\partial T}{\partial t} = K_z \frac{\partial^2 T}{\partial z^2} - \frac{C_{pg}\varepsilon_b}{R}\frac{\partial}{\partial z}(vP) - C_{p,a}\rho_{b,ads} T \sum_{i\in I}\frac{\partial \overline{q}_i}{\partial t} \\ - \frac{C_{pg}\varepsilon_t}{R}\frac{\partial P}{\partial t} + \rho_{b,ads}\sum_{i\in I}(-\Delta H_i)\frac{\partial \overline{q}_i}{\partial t} - \frac{2h_{in}}{r_{in}}(T - T_w),\end{aligned} \tag{S1d}$$

where, $C_{p,ads}$ and $C_{p,a}$ are respectively the adsorbent and adsorbate heat capacity, K_z is the axial heat conductivity, C_{pg} is the gas mixture heat capacity, ΔH_i is the heat of adsorption of component i, h_{in} is the column-wall heat transfer coefficient, and r_{in} is the bed column radius.

The following steady-state momentum balance, i.e., Darcy's law is used for taking into account the pressure drop along adsorbent column:

$$-\frac{\partial P}{\partial z} = \frac{150}{4r_p^2}\left(\frac{1-\varepsilon_b}{\varepsilon_b}\right)^2 \mu v, \tag{S1e}$$

where, r_p is the particle radius and μ is the gas mixture viscosity.

To reduce the computational complexity of capturing mass transfer of adsorbate from gas to solid phase and vice versa, the linear driving force (LDF) model is used

$$\frac{\partial \overline{q}_i}{\partial t} = k_i (q_i^* - \overline{q}_i), \tag{S1f}$$

where, q_i^* is the equilibrium adsorption capacity that is computed using dual-site Langmuir isotherm, and k_i is the LDF mass transfer coefficient.

MOC Process Performance Metrics

The oxygen purity (P_{O_2}) is obtained for the production step of the PSA cycle by calculating the amount of oxygen at the product outlet divided by the total amount of oxygen and nitrogen as follows:

$$P_{O_2}(\%) = \frac{\left(\int_0^{t_f} \frac{y_{O_2}\overline{P}P_0\bar{v}v_0}{R\overline{T}T_0}\Big|_{Z=1} dt\right)}{\left(\int_0^{t_f} \frac{y_{O_2}\overline{P}P_0\bar{v}v_0}{R\overline{T}T_0}\Big|_{Z=1} dt\right) + \left(\int_0^{t_f} \frac{y_{N_2}\overline{P}P_0\bar{v}v_0}{R\overline{T}T_0}\Big|_{Z=1} dt\right)} \times 100, \tag{S2a}$$

where, $Z = 1$ denotes the product outlet end, y_{O_2} and y_{N_2} are the gas phase compositions of oxygen and nitrogen, $\overline{P}$, $\bar{v}$ and $\overline{T}$ are respectively the scaled pressure, interstitial velocity and temperature, P_0, v_0 and T_0 are the respective scaling parameters, and t_f is the production step duration of a PSA cycle.

The net oxygen production amount is calculated by subtracting the amount of oxygen used during purge and pressurization steps from the amount of oxygen obtained during the production step. Using this, the overall oxygen recovery (R_{O_2}) of a PSA cycle is derived as follows wherein the denominator represents the amount of fresh oxygen fed during the production step:

$$R_{O_2}(\%) = \frac{\left(\int_0^{t_f} \frac{y_{O_2}\overline{P}P_0\bar{v}v_0}{R\overline{T}T_0}\Big|_{Z=1} dt\right) - \left(\frac{y_{p,O_2}P_p v_p t_p}{RT_p}\right) - \left(\int_0^{t_{pres}} \frac{y_{pres,O_2}\overline{P}P_0\bar{v}v_0}{RT_{pres}}\Big|_{Z=pres.\ inlet} dt\right)}{\left(\frac{y_{f,O_2}P_f v_f t_f}{RT_f}\right)} \times 100, \tag{S2b}$$

where, y_{p,O_2}, y_{pres,O_2} and y_{f,O_2} are respectively the oxygen molar fraction of purge, pressurization and production feed streams, T_p, T_f and T_{pres} are the purge, production and pressurization step feed temperatures, t_p and t_f are the duration for purge and production steps, and $Z = pres.\ inlet$ is the inlet column end during pressurization step. In addition, v_p, v_f and P_p, P_f are respectively the interstitial feed velocity and pressure for purge and production streams.

To calculate the standard amount of oxygen production rate (PC_{O_2}), the net number of moles of oxygen collected during production step of a PSA cycle is converted to L/min at standard conditions as follows:

$$PC_{O_2}\ (\text{L/min}) = \left[\left(\int_0^{t_f} \frac{y_{O_2}\overline{P}P_0\bar{v}v_0}{R\overline{T}T_0}\Big|_{Z=1} dt\right) - \left(\frac{y_{p,O_2}P_p v_p t_p}{RT_p}\right) - \left(\int_0^{t_{pres}} \frac{y_{pres,O_2}\overline{P}P_0\bar{v}v_0}{RT_{pres}}\Big|_{Z=pres.\ inlet} dt\right)\right] \times \left(\frac{RT^{STP} \times 1000 \times 60}{P^{STP}t_{cycle}}\right), \tag{S2c}$$

where, T^{STP} = 273 K and P^{STP} = 101325 Pa are the standard temperature and pressure conditions, and t_{cycle} is the duration of a PSA cycle in seconds.

Finally, we compute the BSF in terms of amount of adsorbent required in kg to produce 1 ton per day of net oxygen product as follows:

$$\text{BSF} = \frac{\rho_{b,ads}\pi r_{in}^2 L}{\left[\left(\int_0^{t_f} \frac{y_{O_2}\overline{P}P_0\bar{v}v_0}{R\overline{T}T_0}\Big|_{Z=1} dt\right) - \left(\frac{y_{p,O_2}P_p v_p t_p}{RT_p}\right) - \left(\int_0^{t_{pres}} \frac{y_{pres,O_2}\overline{P}P_0\bar{v}v_0}{RT_{pres}}\Big|_{Z=pres.\ inlet} dt\right)\right]} \times \left(\frac{1000 \times t_{cycle}}{24 \times 3600 \times 0.032}\right), \tag{S2d}$$

where, $\rho_{b,ads}$ is the adsorbent bulk density, and r_{in} and L are the column radius and length, respectively.

Decision Variables Bounds for Process Optimization

Table S1. Decision variable bounds on design and operation of adsorption-based MOC unit.

Input variable(s)	Unit	Lower bound	Upper bound
Feed flow rate	mol/s	0.01	0.25
Step pressure	bar	-3.5*	3.5
Step duration	s	1	10
Purge flow velocity factor	-	0.1	3
Adsorbent bulk density	kg ads./m^3 bed	0.35 $\rho_{p,ads}$	0.65 $\rho_{p,ads}$

*negative pressure is an abstract concept to optimize flow direction.

Process Simulation Parameters

Table S2. Parameters utilized for solving the NAPDE-based process simulation model.

Input variable(s)	Unit	Value
Axial gas heat conductivity	J/(m·s·K)	0.29
Bed length	m	0.127
Bed radius	m	0.05
Bed-wall heat transfer coefficient	W/(m^2·K)	70
Feed temperature	K	298
Number of cycles	-	50
Number of spatial nodes	-	10
N_2 viscosity	Pa · s	1.78×10^{-5}
O_2 viscosity	Pa · s	2.02×10^{-5}
Particle diameter	m	0.035
Wall heat capacity	J/(kg·K)	502
Wall density	kg/m^3	7800
